# Support vector machines for spike pattern classification with a leaky integrate-and-fire neuron

**DOI:** 10.3389/fncom.2012.00078

**Published:** 2012-11-19

**Authors:** Maxime Ambard, Stefan Rotter

**Affiliations:** ^1^Bernstein Center Freiburg, University of FreiburgFreiburg, Germany; ^2^Faculty of Biology, University of FreiburgFreiburg, Germany

**Keywords:** machine learning, synaptic kernel, supervised learning rule, Tempotron, linear separation

## Abstract

Spike pattern classification is a key topic in machine learning, computational neuroscience, and electronic device design. Here, we offer a new supervised learning rule based on Support Vector Machines (SVM) to determine the synaptic weights of a leaky integrate-and-fire (LIF) neuron model for spike pattern classification. We compare classification performance between this algorithm and other methods sharing the same conceptual framework. We consider the effect of postsynaptic potential (PSP) kernel dynamics on patterns separability, and we propose an extension of the method to decrease computational load. The algorithm performs well in generalization tasks. We show that the peak value of spike patterns separability depends on a relation between PSP dynamics and spike pattern duration, and we propose a particular kernel that is well-suited for fast computations and electronic implementations.

## 1. Introduction

Spike pattern classification has become an important topic in several fields of research. For example, electrophysiological recordings from neural networks on Multi-Electrode Arrays (MEA) provide spatio-temporal spike patterns that remain difficult to understand. In those setups, spike pattern classification algorithms are useful to understand how spontaneous network bursting activities that propagate through the neural network are triggered in particular places (Kermany et al., 2010). Experimental evidence tends to show that precise spike timing plays a significant role in the encoding of sensory stimuli (Bohte, 2004; VanRullen et al., 2005), and theoretical considerations have shown that spiking neuron models can have better computational capabilities for fast information processing than firing rate based neuron models (Maas, 1997). Understanding how synaptic weights determine the neuronal input–output function is one of the most important challenges of computational neuroscience. Electronic devices based on temporal pulse coding have interesting properties for sensors and neuroprosthesis development (Boahen, 2002; Ambard et al., 2008; Chen et al., 2011), and recent research on novel computer architectures focuses on low-power spike based computing chips (Modha, 2011).

Previous methods for spike pattern classification can be separated into two families. The first family uses spike train metrics (for an overview, see Brown et al., 2004; Grün and Rotter, 2010). Some of these methods are based on arbitrary features extracted from the observed spike patterns such as rank order (Pan et al., 2009), firing rate (Kermany et al., 2010), or inter-spike intervals (ISI) (Abeles and Gerstein, 1988; Christen et al., 2004). These methods run the risk of preselecting features that are not relevant for biology. Others use metrics based on vector-space embeddings (van Rossum, 2001; Schrauwen and Campenhout, 2007; Houghton and Sen, 2008). While those methods are well-known and show good performance in classification tasks, their results are difficult to understand in terms of neural mechanisms. The second family is bio-inspired. It uses neuron models and sets appropriate synaptic weights. Classification is done with a supervised learning rule to control the neural response for a given set of spike patterns (Bohte et al., 2000; Gütig and Sompolinsky, 2006; Kasińnski and Ponulak, 2006; Voegtlin, 2007; McKennoch and Voegtlin, 2009; Urbanczik and Senn, 2009). Those methods are particularly interesting since their results can be easily linked to biological processes.

In this paper, we present a method related to both families of methods. It is based on spike train kernel convolution in the time domain, in conjunction with Support Vector Machines (SVM) to compute an optimal linear decision policy. Coefficients of the corresponding hyperplane can be interpreted as synaptic weights on the dendrite of a leaky integrate-and-fire (LIF) neuron model. In section 2 we describe our new method. We compare its generalization performance with other learning rules sharing the same conceptual framework. We show a relation between spike pattern separability and postsynaptic potentials (PSP) kernel dynamics, and we introduce kernels optimized for fast computation. Section 3 describes in detail the methods used in the simulations. In section 4 we discuss limitations and possible improvements of our approach.

## 2. Results

### 2.1. Support vector machine using postsynaptic potential kernels

Trying to understand how spike patterns are discriminated in biological neural networks by selecting features or by mapping spike trains in *ad-hoc* vector spaces requires assumptions on relevant features used by neural information processing. Another method takes a neuron's point of view by modeling neuronal information processes and letting the neuron figures out which information of spike patterns is relevant. One of the major aspects of the neural processing is the temporal integration of postsynaptic excitatory and inhibitory events that results in a temporal neural response expressed by action potentials. In this scenario, the neural input–output function is determined by the modulation of synaptic event amplitudes, called synaptic weights in numerical models.

The present section describes a new supervised learning rule that optimizes synaptic weights for robust spike patterns classification. Although we would not characterize this method as bio-mimetic, it is related to a common LIF neuron model. The results, that can be considered as optimal synaptic weights, provide new insight on what should be the outcome of biological synaptic plasticity processes for robust spike patterns classification. In the present paper, we refer to this method using the acronym SVM-PSP for Support Vector Machine using Post-Synaptic Potential kernels.

**Figure 1:**
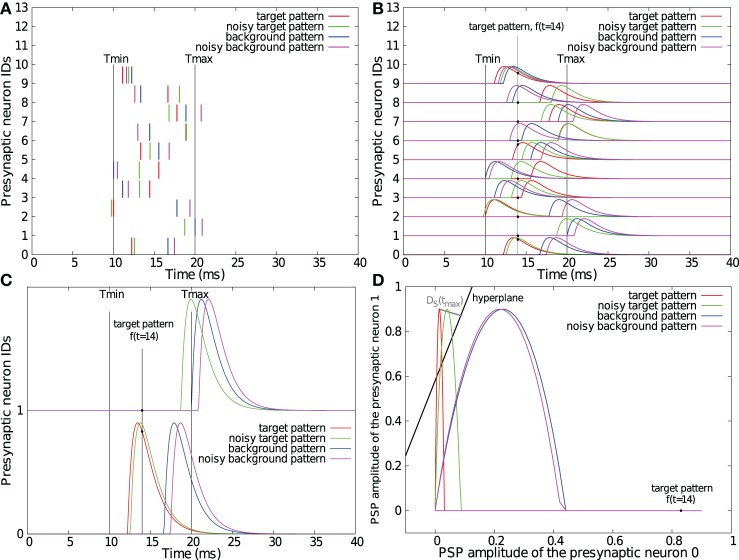
**Illustrations of the SVM-PSP method.** One target and one background spike pattern, superimposed with their noisy version (standard deviation of the Gaussian noise = 1 ms) as generated for section 2.2 **(A)**. Convolution of the spike patterns with a double exponential kernel with τ_*r*_ = 1 ms and τ_*d*_ = 1.5 ms. Note that the maximal PSP amplitude has been fixed to 0.9 for illustration purposes **(B)**. Convolution of spike patterns with two input neurons **(C)** and representation of their trajectories *f*(*t*) in the 2-dimensional feature space *S* superimposed with the computed separating hyperplane **(D)**. Note that orthogonal projection *D*_*S*_(*t*_max_), representing the maximal *D*^+^_*S*_ distance onto the hyperplane, does not appear orthogonal due to different horizontal and vertical axis scalings.

We consider two classes of spike patterns *P*^+^ = {*p*^+^} (target patterns) and *P*^−^ = *p*^−^ (background patterns) that have to be separated from each other. Each spike pattern is composed of incoming spikes from *N* neurons. We define *t*_*ij*_ as the time of the *j*th spike of the *i*th neuron. Let *k*(*t*) be a fixed kernel. For each time *t* we consider the vector *f*(*t*) = (*f*_1_(*t*), *f*_2_(*t*), …, *f*_*N*_(*t*)) where $f_{i}(t)=\sum_{j}k(t-t_{ij})$. Evaluating this function at discrete points in time *t*_*l*_ = Δ*l*, a pattern *p* is transformed into a set of points *f*_*p*_ = {*f*(*t*_1_)} residing in an *N*-dimensional feature space *S* = ℝ^*N*^. Let us denote *F*^+^ = {*f*^+^} (resp. *F*^−^ = {*f*^−^}) the set of points associated to all *p*^+^ (resp. *p*^−^) patterns (see Figures 1A–D for illustration).

The aim of our classification is to find a hyperplane *H*(*W*, *b*) given by the equation *W*_1_ *f*_1_ + *W*_2_ *f*_2_ + … + *W*_*N*_ *f*_*N*_ − *b* = 0 in *S* that separates at least one point *f*^+^(*t*_*l*_) of each *f*^+^ from all the points *f*(*t*_*l*_) of all *f*^−^ as described by the following equations

(1) $$\forall p\in P^{+},\exists t_{l}:\sum_{i}W_{i}\sum_{i}k(t_{l}-t_{ij})-b\geq 0$$

(2) $$\forall p\in P^{-},\forall t_{l}:\sum_{i}W_{i}\sum_{j}k(t_{l}-t_{ij})-b<0$$

If we consider that only one pattern has to be detected, the classification task is to find at least one point *f*(*t*_*l*_) of *f*^+^ that can be linearly separated from all the points *f*^−^(*t*_*l*_) in *F*^−^.

Linear SVM (Vapnik, 1995) can be used to find a hyperplane that optimally separates two classes of points. We used the solver L2-regularized L1-loss support vector classification (dual) with a cost parameter of 10, epsilon of 10^−2^ and an enabled bias, provided by the open-source liblinear python/C++ library (Fan et al., 2008).

Before computing the separating hyperplane, all the points are re-scaled such that the minimal value on each dimension is 0 and the maximal value is 1. To get the separation hyperplane, when only 1 *p*^+^ has to be separated, all the *f*^−^(*t*_*l*_), *p* ∈ *P*^−^ are considered as points that should remain below a hyperplane, and all the points *f*^+^(*t*_*l*_) are tried successively as point that should remain above this hyperplane.

Thus, a new separation hyperplane *H*_*l*_ is computed by the SVM for each tested *f*^+^(*t*_*l*_) and the aim is to find *H*_*l*_ that can separate one *f*^+^(*t*_*l*_) from all the *f*^+^(*t*_*l*_) points with the largest margin. To find this separation hyperplane, let us first consider the signed orthogonal distance between a point *f*(*t*_*l*_) and a hyperplane *H* given by

(3) $$D_{S}(H,f(t_{l}))=\frac{\langle W,f(t_{l})\rangle -b}{\left\|W\right\|}$$

For each tested *f*^+^(*t*_*l*_) point, we compute *D*^+^_*S*_ = *D*_*S*_(*H*_*l*_, *f*^+^(*t*_*l*_)), its distance from the corresponding hyperplane *H*_*l*_. The minimal distance between all the *f*^−^(*t*_*l*_) points and the hyperplane is also computed and is given by

(4) $$D_{S}^{-}=-\max(D_{S}(H_{l},f^{-}(t_{l})))$$

The ability of the hyperplane *H*_*l*_ to separate the *f*^+^(*t*_*l*_) point from all the *f*^−^(*t*_*l*_) is given by

(5) $$D_{S}(H_{l},f^{+}(t_{l}),P^{-})=\min(D_{S}^{+},D_{S}^{-})$$

*D*_*S*_(*H*_*l*_, *f*^+^(*t*_*l*_), *P*^−^) is successively computed for all *f*^+^(*t*_*l*_) ∈ *p*^+^. The hyperplane *H*_*l*_ leading to the maximal margin *D*_*S*_(*p*^+^, *P*^−^) is considered as the result of our method.

(6) $$D_{S}(p^{+},P^{-})=\max(D_{S}(H_{l},f^{+}(t_{l}),P^{-}))$$

To compute a normalized separability measure that is independent of the number of dimensions of the space *S* (the number of input neurons), we compute $D_{N}(p^{+},P^{-})=2D_{S}(p^{+},P^{-})/\sqrt{N}$ where $\sqrt{N}/2$ is the maximal separation margin between two points in normalized feature space [0,1]_*N*_.

Although this learning rule is not bio-mimetic and takes place in a high-dimensional feature space, its result is directly related to LIF neuron parameters. Hyperplane coefficients *W* can be transformed into synaptic weights *W* by multiplying them by θ/*b* where θ is the LIF spike threshold potential. After the best separation hyperplane has been found, the dot product between the vector θ/*b W* and the vector *f*(*t*_*l*_) is equivalent to the LIF membrane voltage at time *t*_*l*_. The result of this method keeps all the points *f*^−^(*t*_*l*_) of all background patterns below the hyperplane and at least one point *f*^+^(*t*_*l*_) of the target pattern above this hyperplane. Therefore, it is equivalent to fixing synaptic weights that constraint the LIF membrane voltage below the threshold potential for background patterns, ensuring that membrane voltage exceeds the threshold at least once for the target pattern. Thus, the neuron fires only when the learned target pattern is received. However, using SVM to maximize *D*_*S*_ not only fixes a separation hyperplane, but it also improves the classification reliability against noisy spike patterns. This property is shown in the next section.

### 2.2. Using SVM improves generalization performance

To quantify the robustness of our method (see section 2.1) against noisy spike patterns, we compared its generalization performance against the original Tempotron algorithm (Gütig and Sompolinsky, 2006 see section 3.2), and a slightly modified Tempotron learning rule that we call the voltage-margin Tempotron (explained in section 3.3).

We used the double exponential *k*(*t*) = *e*^−*t*/1.5^ − *e*^−*t*^ as kernel function. Five negative patterns and one positive pattern were generated (see section 3.1). Time between 0 and 40 ms was discretized with a period of 0.1 ms. For each noise level, the simulation was repeated over 100 trials. In each trial, 100 noisy patterns were created from each learned pattern (i.e., 100 noisy target patterns, 500 noisy background patterns) and used for testing. The three hyperplanes (*H*_SVM_, *H*_Temp_, and *H*_Temp_M__, respectively) found by applying the SVM-PSP, the Tempotron and the voltage-margin Tempotron algorithms were tested on a generalization task on same noisy spike patterns (see section 3.1).

**Figure 2:**
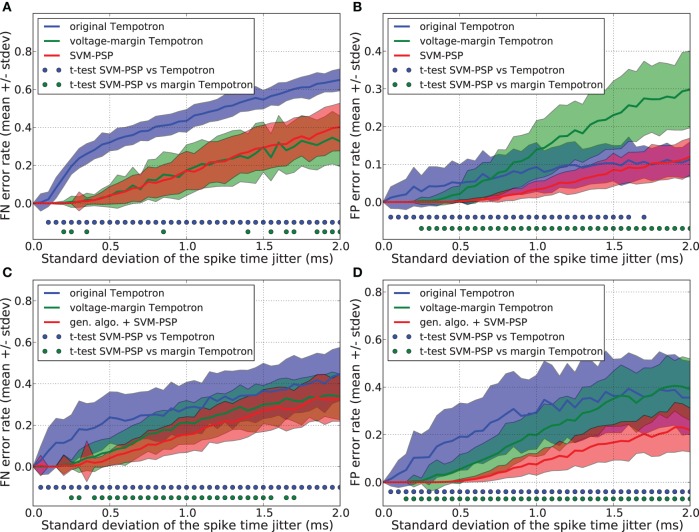
**Generalization performance measured as the number of misclassified noisy patterns over the total number of tested noisy patterns.** Comparison between the original Tempotron, a voltage-margin Tempotron, and SVM-PSP algorithm with 10 presynaptic neurons. Mean value and standard deviation of false negative (FN) error **(A)** and false positive (FP) error **(B)**. The learning task is to separate one target pattern from 5 background patterns. Hundred jittered variants were generated from each learned pattern and used for testing the generalization abilities of our method. For each noise level, 100 trials were then performed. Mean value and standard deviation of false negative (FN) error **(C)** and false positive (FP) error **(D)**. The learning task is to separate 2 target patterns from 4 background patterns. Blue (resp. green) dots denote a significant difference of the generalization results (paired *t*-test, *p* < 0.05) between our method and the original Tempotron (resp. voltage-margin Tempotron).

Figures 2A,B show that the original Tempotron learning rule leads to a false negative (FN) error rate of about 30% larger than the voltage-margin Tempotron and the SVM-PSP, while it has a low false positive (FP) error rate. As explained in section 3.2, the original Tempotron learning rule stops when a separation has been found without trying to optimize it. This causes generalization performance limitations, especially with regard to FN error when the synaptic weights are initialized to 0, leading to minimal synaptic weights. This explains why, even for small noise, FN error is large and why FP error is small.

Voltage-margin Tempotron and SVM-PSP learning rules have a similar FN error-rate. The SVM-PSP algorithm provides better FP error rate than the other two algorithms, except for the case of strong noise, where the original tempotron and the SVM-PSP perform similarly. The voltage-margin Tempotron overcomes the synaptic weights initialization problem by increasing the voltage margin between points relatively to the voltage threshold, but results are still lower than SVM-PSP. This shows that maximizing *D*_*S*_ is better than *D*_*V*_ (see sections 2.1 and 3.3) in improving generalization results.

However, while the Tempotron learning rule does not require many changes when the task is to separate several *p*^+^ patterns instead of one, this is more problematic for the SVM-PSP method. Separating *n p*^+^ patterns with this method would require testing all possible *n*-tuples [*f*^+^_1_(*t*_*l*_1__), …, *f*^+^_*n*_(*t*_*l*_*n*__)]. Due to combinatorial explosion, it is practically impossible to compute all hyperplanes to find the one leading to the highest separation. Genetic algorithms offer a good solution to this problem when dealing with optimization of non-linear functions in which parameters are subject to combinatorial explosion.

Generalization performance was compared for the separation hyperplanes found with a genetic algorithm combined with the SVM-PSP method (see section 3.4), an original Tempotron (see section 3.2) and a voltage-margin Tempotron (see section 3.3). This was performed for a task consisting in separating 2 *p*^+^ from 4 *p*^−^ patterns. To prevent promoting one algorithm against another in terms of learning cycles, we took the number of hyperplanes calculated by the voltage-margin Tempotron learning rule as the maximum number of genotypes tested by the genetic algorithm.

Figures 2C,D show the FN and FP error rates. Generalization performance is lower both for the original Tempotron and the voltage-margin Tempotron learning rule as compared to the SVM-PSP method associated with a genetic algorithm. This is especially relevant for FP errors. The original Tempotron has a more symmetric error profile compared to Figure 2A. Adding a second target pattern eliminates the influence of null synaptic weights initialization (see section 3.2). The error profile of the SVM-tempotron method is also more symmetric, but FN error rate remains larger than FP error rate. Since, compared to *p*^−^ patterns, the separation hyperplane is built on few *p*^+^ points that are most probably situated on an extremity of trajectories, they could be more subjected to variation when jitter is added to the spike pattern. The small error rate for the SVM-PSP method shows that maximizing the separation margin in the feature space provides good generalization performance although not all possible hyperplanes have been tested.

**Figure 3:**
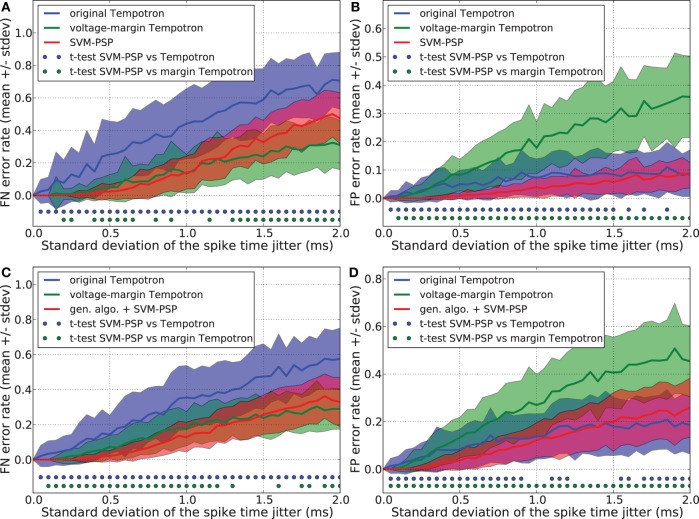
**Generalization performance measured as the number of misclassified noisy patterns over the total number of tested noisy patterns.** In learned patterns, each neuron fires once at a time that is uniformly distributed in the interval [10 ms, 20 ms]. Comparison between the original Tempotron, a voltage-margin Tempotron, and SVM-PSP algorithm with 10 presynaptic neurons. Mean value and standard deviation of false negative (FN) error **(A)** and false positive (FP) error **(B)**. The learning task is to separate one target pattern from 5 background patterns. Hundred jittered variants were generated from each learned pattern and used for testing the generalization abilities of our method. For each noise level, 100 trials were then performed. Mean value and standard deviation of false negative (FN) error **(C)** and false positive (FP) error **(D)**. The learning task is to separate 2 target patterns from 4 background patterns. Blue (resp. green) dots denote a significant difference of the generalization results (paired *t*-test, *p* < 0.05) between our method and the original Tempotron (resp. voltage-margin Tempotron).

We compared the generalization performance between our new method and the others, based on spike patterns with variable ISIs. With this new pattern generation procedure, each neuron fires once at a time that is uniformly distributed in the interval [10 ms, 20 ms]. The same analysis as above was performed and results are shown in Figure 3. Whereas the SVM-PSP method has higher FN error rate than the voltage-margin Tempotron for strong noise, our method presents the best global generalization performance (FN + FP) both in separating 1 *p*^+^ from 5 *p*^−^, and 2 *p*^+^ from 4 *p*^−^ patterns.

**Figure 4:**
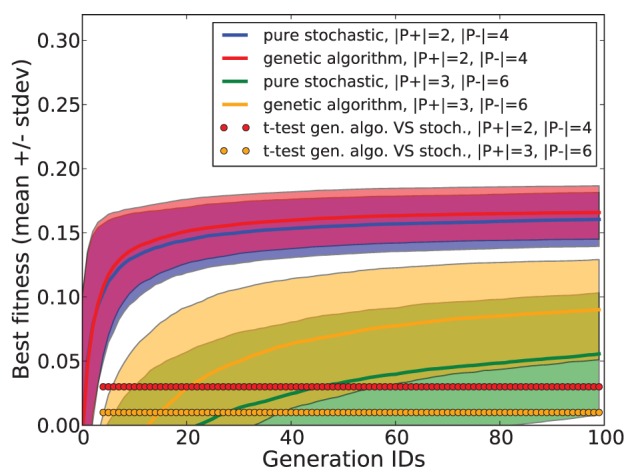
**Fitness performances obtained with a genetic algorithm and with a pure stochastic search in tasks consisting in separating 2 *p*^+^ patterns from 4 *p*^−^ patterns and separating 3 *p*^+^ from 6 *p*^−^ patterns**.

Employing additional simulations, we checked the benefit of using a genetic algorithm compared to a pure stochastic search where both reproduction and mutation are disabled. In this stochastic search, each new generation is composed of random specimen. We measured the best fitness obtained after each generation for both methods, using a task consisting in separating 2 *p*^+^ patterns from 5 *p*^−^ patterns, and for another task consisting in separating 3 *p*^+^ patterns from 6 *p*^−^ patterns. The simulation was repeated for 1000 trials. Mean and standard deviation of the best fitness are shown in Figure 4. Statistical testing was done using a paired *t*-test for each generation. Compared to a pure stochastic search, the use of a genetic algorithm guarantees a significant improvement which increases with task difficulty.

These results show that using SVM to find a separation hyperplane provides better generalization results than using the Tempotron learning rule, while keeping the same bio-inspired model (i.e., synaptic weights for a simple integrate-and-fire spiking neuron model). Although the use of SVM ensures an optimized linear separation in the feature space *S*, the shape of the kernel, in particular its time constant, is important in the pre-mapping of spike patterns in the feature space. The next section study this relation.

### 2.3. Spike pattern separability depends on shape of postsynaptic potentials

To know how dynamics of the kernel affect the performance of the method, we tested spike pattern separability (see section 2.1) with different kernel functions and various time constants.

**Figure 5:**
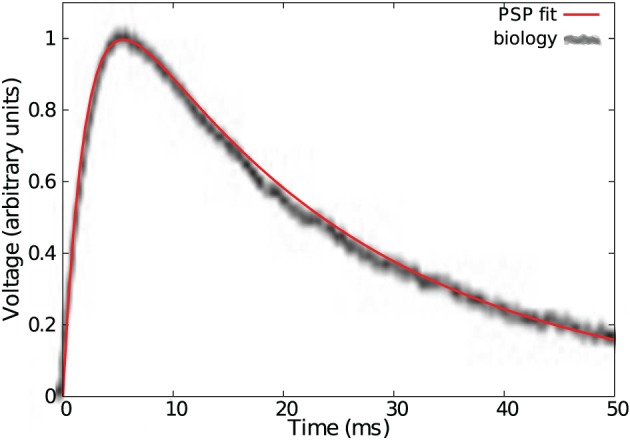
**Fit of a typical cortical EPSP with a double exponential bio-mimetic kernel (τ = τ_*d*_ = 23 ms, τ_*r*_ = 0.09τ)**.

Three kernel functions were compared: a single exponential of equation *e*^−*t*/τ^, an α-function *t*/τ *e*^−*t*/τ^ and a double exponential kernel *e*^−*t*/τ^−*e*^−*t*/τ_*r*_^ fitted from Povysheva et al. (2006) where the rising time constant τ_*r*_ is set to 0.09 τ (see Figure 5).

Spike patterns were generated by the procedure explained in section 3.1. The time constant τ was varied between 0.25 and 64 ms. Time between 0 ms and τ + 10 ms was discretely sampled with resolution of 0.1 ms. The best hyperplane was chosen according to the method described in section 2.1. We used the normalized distance *D*_*N*_ (see section 2.1) to obtain a measure for patterns separability. We write ν = τ/*T* for the time constant normalized for pattern duration, *t* being the period of learned spike patterns. Maximal distance in the feature space between the trajectory and the synchrony vector *f*_*s*_ = (1, 1, …, 1) was quantified by the measure *L*_*s*_ (see Appendix A.1).

**Figure 6:**
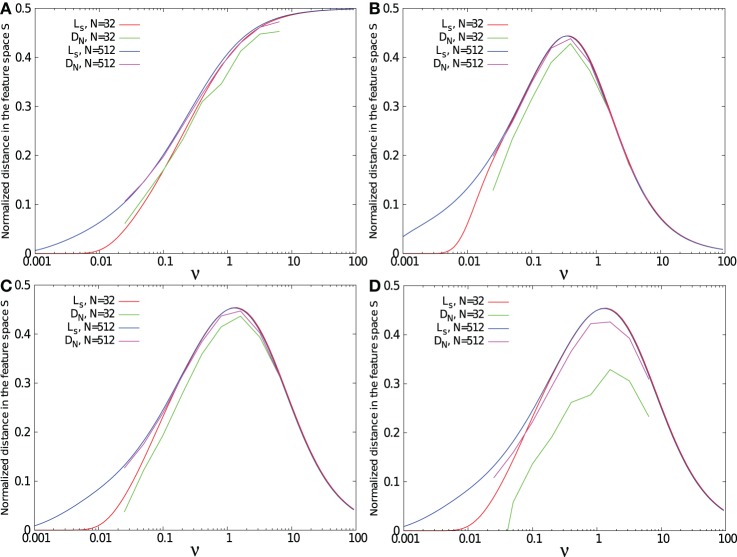
**Separability measure *D*_*N*_(*t*_max_) and maximal standard deviation *L*_*s*_ as function of the relative time constant of the kernel ν = τ/*T*.** Results were obtained for a task consisting in separating 1 target pattern from 1 background pattern except in **(D)** where the task was to separate 1 target pattern from 20 background patterns. Simulations were performed using 32 and 512 input neurons, with a single exponential kernel **(A)**, with a α kernel **(B)**, and with a bio-mimetic kernel **(C)** and **(D)**.

Figures 6A–C show the separability for the three kernels, each with spike patterns coming from either 32 or 512 afferent neurons. The task was to separate 1 *p*^+^ pattern from 1 *p*^−^ pattern.

For the three tested kernels, separability is low for small ν. Due to the small time constant τ compared to the mean ISI of the spike pattern defined by *T*/*N*, all trajectories *f*(*t*) fluctuate in the regime of asynchronous inputs, spanned by asynchrony base *B*_*A*_ = [(0, 0, …, 0), (1, 0, …, 0), …, (0, 0, …, 1)]. Therefore, *f*^+^ and *f*^−^ trajectories remain close to each other, leading to a low separability. Increasing the number of presynaptic neurons from 32 to 512 improves the spike pattern separability, since the mean ISI decreases with the number of afferent neurons. As a result, for a given PSP time constant, the neuron model is better at separating long lasting spike patterns when the number of afferent neurons is large.

Separability for both the α and the double exponential kernels decreases after a peak for intermediate ν values. For large ν values, all *f*(*t*) trajectories converge toward the synchrony vector *f*_*s*_, due to the slow kernel dynamics compared to the ISI of the pattern. At this range of ν values, increasing the number of presynaptic neurons does not affect separability. As a consequence, for all ν values, separability between two spike patterns is better with a large number of afferent neurons.

However, separability with the single exponential kernel increases monotonically with ν. The single-exponential kernel has a discontinuity bringing it instantly from 0 to its maximal value when a spike is received. For large time constants, this kernel becomes similar to a step function. For this reason, the trajectory does not lie on the synchrony vector, but goes from one corner of feature space to another. It reaches the synchrony point *f*_*s*_ when all spikes have been received.

For a simple task such as separating two patterns from each other, separability is closely related to the distance *L*_*s*_ between trajectories and the synchrony vector, especially for large number of presynaptic neurons and large time constants. However, as shown in Figure 6D, increasing the number of background patterns to 20 decreases separability, especially when the number of presynaptic neurons is small. For large enough number of afferent neuron compared to the number of background patterns, the volume of the feature space [0,1]^*N*^ is wide enough to provide separability close to *L*_*s*_. When the number of background patterns increases, the space becomes more “populated” and separability decreases.

Whatever the number of afferent neurons and the number of spike patterns are, the value of ν leading to the separability peak remains the same and can directly be fixed by searching for kernel dynamics that maximize *L*_*s*_. Therefore, using the measure *L*_*s*_ can give a fast and simple method to properly fix a kernel shape that maximizes spike patterns separability.

Note in Figure 6C that a bio-mimetic kernel has its separability peak value for ν_max_ = 1.3. Therefore, maximal pattern separability with a time constant of τ = 23 ms (see Figure 5) occurs with a spiking period of *T* = τ ν_max_ = 17.7 ms, that corresponds to the low range (≈0.50 Hz) of the so-called (low) γ frequency band (Fries, 2009).

The choice of the PSP kernel function is a key step in designing the algorithm for spike pattern classification. Both too narrow and too wide kernels lead to sub-optimal separability. For one and the same kernel, a neuron receiving input from a larger number of presynaptic neurons is better in separating long-lasting spike patterns. The relation between kernel dynamics and pattern duration for spike pattern classification has already been emphasized using different methods in Rubin et al. (2010).

### 2.4. PSP kernels for fast computation with the SVM-PSP method

**Figure 7:**
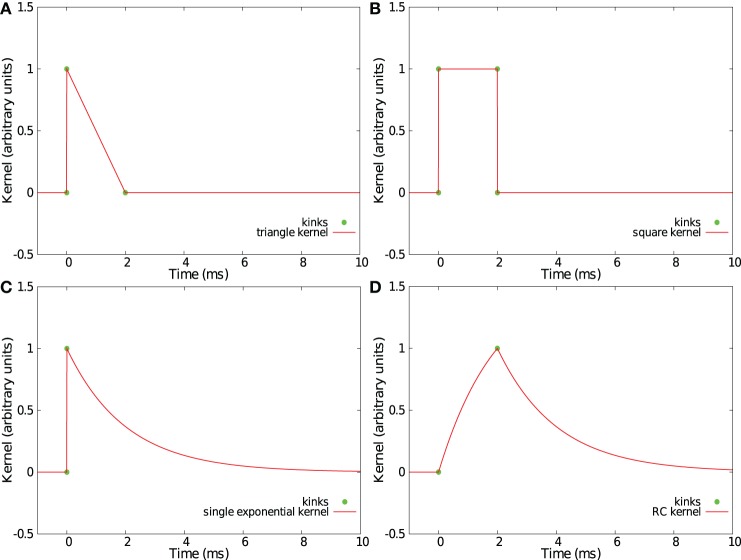
**Examples of kernels leading to piecewise linear trajectories in feature space.** Shown are a triangular kernel **(A)**, a square kernel **(B)**, a single exponential kernel **(C)**, and a RC-kernel **(D)**.

Convolving incoming spikes with an α-function or a double exponential kernel, such those as presented in sections 2.2 and 2.3, leads to curved trajectories in the feature space *S*. Thus, with our method, every point in the trajectory makes a relevant contribution to find the best separation hyperplane, so a fine temporal discretization of the trajectories *f*(*t*) is helpful. However, some PSP kernels have an acceleration vector $A(t)=\ddot{f}(t)$ equal to zero or collinear with the speed vector *V*(*t*) = ḟ(*t*) except at some particular times where the derivative of the kernel is not continuous. Those kernels produce piecewise linear trajectories in the space *S* with kinks corresponding to discontinuities of the derivative. In such trajectories, only the points of discontinuity need to be considered as relevant points when computing the separation hyperplane. Compared to the case of *L* equidistant samples as used in sections 2.2 and 2.3 for which a parameter Δ requires to be fixed, this method provides a more simple and more effective set of points. Four such kernels already considered in Rotter and Diesmann (1999) are shown in Figures 7A–D.

We compared the generalization performance between the double exponential kernel used in section 2.3, a single exponential kernel considered in section 2.3, and a “RC kernel” with a time evolution described by the following equations.

$$k_{RC}(t)=\{\begin{array}{l} (1-e^{-t/\tau }),\;0\leq t\leq T_{\text{pulse}}\\ (1-e^{-T_{\text{pulse}}/\tau })e^{-(t-T_{\text{pulse}})/\tau },\;t\geq T_{\text{pulse}}\; \end{array}$$

*T*_pulse_ denotes the time where the double exponential function reaches its maximal value (i.e., *T*_pulse_ = τ τ_r_ log(τ/τ_r_)/(τ − τ_r_)). For the three kernels, the time constant was set to τ = 13 ms = 1.3 *T* where *T* = 10 ms is duration of spike patterns. The task was to separate 1 *p*^+^ pattern from 5 *p*^−^ patterns. The number *N* of presynaptic neurons was equal to 10.

**Figure 8:**
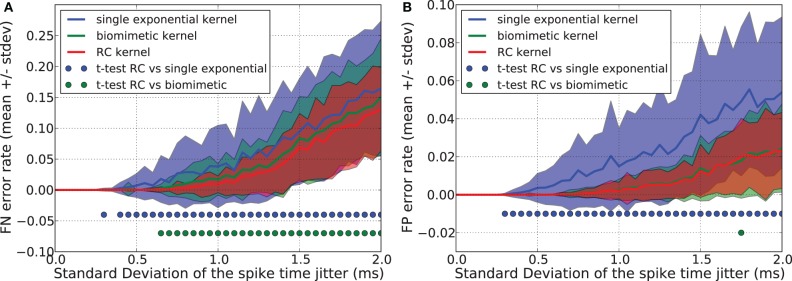
**Generalization performance measured as the number of misclassified noisy patterns over the total number of tested noisy patterns.** Comparison between a single exponential kernel, a RC kernel and a bio-mimetic kernel. The task is to separate one target pattern from 5 background patterns. Mean value and standard deviation of false negative (FN) error **(A)** and false positive (FP) error **(B)**. Blue (resp. green) dots denote a significant difference of the generalization results (paired *t*-test, *p* < 0.05) obtained with the RC kernel and the single exponential kernel (resp. biomimetic kernel).

Figures 8A,B show that the single exponential kernel has lower performance, both with regards to FN and FP errors, than the bio-mimetic and the RC kernel, which have similar generalization error rate. Triangular, square, and single exponential kernels imply linear trajectories but are highly discontinuous, jumping instantaneously from 0 to 1 when a spike occurs. This causes severe problems in generalization task. The RC kernel, in contrast, has some advantages: each spike creates two kinks in the trajectory, the number of points to consider {*f*(*t*_*ij*_, *f*(*t*_*ij*_ + *T*_pulse_))} is thus *N*_*k*_ = 2 *N*_*s*_ where *N*_*s*_ is the number of presynaptic spikes, it provides “smoother” trajectories and, therefore, better generalization performance compared to the three other kernels and it corresponds to a simple and well-known RC circuit that received at time *t* a current pulse of duration *T*_pulse_.

While providing similar generalization results as a curved double exponential kernel, using the RC kernel with the SVM-PSP method allows much faster computation especially for sparse spike patterns.

## 3. Materials and methods

### 3.1. Spike pattern generation and generalization error measure

To generate spike patterns used in learning tasks, the time, *t*_*i*_, of the spike of neuron *i* ∈ {1, …, *N*} in a pattern *p*, is given by *t*_*i*_ = *T*_min_ + (*u*(*p*, *i*) − 1)(*T*_max_ − *T*_min_)/(*N* − 1), where, for each *p*, {*u*(*p*, 1), *u*(*p*, 2), …, *u*(*p*, *N*)} is an independently chosen, random permutation of {1, 2, …, *N*}, which indicates the order in which the neurons fire. *T*_min_ and *T*_max_ are the minimum and the maximum time of spike reception. We used *T*_min_ = 10 ms and *T*_max_ = 20 ms. *T* = *T*_max_ − *T*_min_ denotes the spike pattern period. This is true only if no jitter is present. In the case of jitter, some spikes may come to lie outside of [*T*_min_, *T*_max_]. With this procedure, each spike pattern consist in *N* neuron firing one spike between *T*_min_ and *T*_max_ in a random order with a constant ISI.

To test generalization performance in sections 2.2, and 2.4, random jitter is introduced in each spike *i* of each learned spike pattern *j*. A new spike time t^*^_ij_ is generated by adding a Gaussian noise Noise(0, σ) to each spikes time *t*_*ij*_. If t^*^_ij_ ≤ 0 ms or t^*^_ij_ >30 ms, a new noise value is pulled again.

Noisy *p*^+*^ and *p*^−*^ patterns created from each learned *p*^+^ and *p*^−^ pattern are tested for classification. The simulation is repeated with a noise parameter σ that is varied between 0 and 2 ms.

FN error rate is the number of noisy *p*^+*^ patterns that have not been detected divided by the total number of noisy *p*^+*^ patterns. FP error rate is the number of noisy *p*^−*^ patterns that have been detected divided by the total number of noisy *p*^−*^ patterns.

In section 2.2, the statistical significance of the generalization ability of our method against the Tempotron and the voltage-margin Tempotron were tested with a paired *t*-test. In section 2.4, the same procedure was applied to test the generalization ability of a RC kernel against a bio-mimetic kernel, and a single-exponential kernel.

### 3.2. Original discrete tempotron

The Tempotron algorithm is based on the integrate-and-fire neuron model. The time evolution of the membrane potential *V* is described by the following equation

(7) $$V(t)=V_{\text{rest}}+\sum_{i}w_{\text{i}}\sum_{j}k(t-t_{ij})$$

in which *t* is the time, *V*(*t*) is the membrane potential, *w*_*i*_ is a set of synaptic weights, *t*_*ij*_ is the time of the *j*th spike time of the *i*th presynaptic neuron, *k*(*t*) is a postsynaptic kernel function and *V*_rest_ is the resting potential. *N* is the number of input neurons. The Tempotron is said to detect a spike pattern when its membrane potential exceeds a threshold value θ. For simplicity, we use *V*_rest_ = 0 and θ = 1 in simulations.

The aim of the original Tempotron learning rule is to find an appropriate set of synaptic weights leading the membrane potential *V*(*t*) above the threshold potential θ for spike patterns that should be detected (*p*^+^), and to keep the membrane voltage below the threshold for background spike patterns that should not be detected (*p*^−^). Again, using discrete time *t*_*l*_ = Δ*l*, a pattern *p* is transformed into a set of voltage values *V*_*p*_ = {*V*(*t*_l_)}. The stop condition of this learning rule is described by the following equations

(8) $$\forall p\in P^{+},\exists t_{l}:V(t_{l})\geq \theta$$

(9) $$\forall p\in P^{-},\forall t_{l}:V(t_{l})<\theta$$

For illustration, let us consider a task where two spike patterns, *p*^+^ and *p*^−^, have to be separated. A dot product between the two spike trains convolved with the kernel and those synaptic weights leads to two voltage traces *V*^+^(*t*) and *V*^−^(*t*) as described in Equation 7. If the maximum value of V^−^(*t*) occurs at *t*_*l*_ = *t*^−^_max_ and *V*^−^(*t*^−^_max_) = *w*_1_*V*_1_(*t*^−^_max_) + *w*_2_*V*_2_(*t*^−^_max_) + ··· + *w*_*N*_*V*_*N*_(*t*^−^_max_) > θ, there is a misclassification. In this case, the Tempotron learning rule consists in the modification of all synaptic weights *w*_*i*_ by subtracting an amount of $\Delta_{{\text{w}}_{\text{i}}}=\lambda \sum_{j}k(t_{\max}-t_{ij})$, λ being a learning coefficient. The same procedure is applied when *V*^+^(*t*^+^_max_) < θ, except that Δ_*w*_*i*__ is added to the synaptic weights. In this paper, synaptic weights are initialized to 0 and the λ parameter is equal to 0.1.

From a geometrical point of view as explained in section 2.1, fixing synaptic weights is equivalent to selecting a separation hyperplane according to equation *w*_1_*V*_1_ + *w*_2_*V*_2_ + ··· + *w*_*N*_*V*_*N*_ − θ = 0, where θ is the bias of the hyperplane. Checking if there is a *t*_*l*_ for which *V*^−^(*t*_*l*_) ≥ θ is equivalent to checking if a point *f*^−^(*t*_*l*_) of *F*^−^_*p*_ is above the current hyperplane, and modifying all synaptic weights *w*_*i*_ by an amount of Δ_*w*_*i*__ is equivalent to subtracting the vector λ *f*(*t*^−^_max_) from the vector *w* = (*w*_1_, *w*_2_, …, *w*_*N*_). Thus, the Tempotron and the SVM-PSP method share the same framework. The difference lies in the process of setting the hyperplane coefficients.

### 3.3. Voltage-margin tempotron

To maximize the voltage separation between *p*^+^ and *p*^−^ patterns, the stop condition of the Tempotron algorithm can be modified by adding a voltage margin *M*_*V*_ as described in the following equations

(10) $$\forall p\in P^{+},\exists t_{l}:V(t_{l})\geq \theta +M_{V}$$

(11) $$\forall p\in P^{-},\forall t_{l}:V(t_{l})<\theta -M_{V}$$

The algorithm starts with a margin *M*_*V*_ = 0. When a separative set of synaptic weights has been found, the margin is increased by an amount of Δ_*M*_*V*__ that is fixed (we use Δ_*M*_*V*__ = 0.01). The Tempotron learning rule is successively applied with an increasing margin. This process is repeated until a larger margin cannot be reached anymore. It is hard to decide whether a broader margin cannot be reached any more or if the algorithm requires more time to find it (except when the margin is equal to θ which is the highest margin that can be reached, the origin point *O* = (0, 0, …, 0) being in each *p*^−^ pattern). For this reason, the algorithm stops when the learning rule has been applied 100 times successively without finding a separation for a given margin.

Maximizing the measure *D*_*V*_ = *w*_1_*V*_1_ + *w*_2_*V*_2_ + ··· + *w*_*N*_*V*_*N*_ − θ is equivalent to maximizing ‖*w*‖*D*_*S*_ (see Equation 3). Let ρ = θ/‖*w*‖ denotes the length of the orthogonal projection of the origin point *O* onto the separation hyperplane in the feature space *S*. From a geometric point of view, maximizing *D*_*V*_ amounts to finding a separation hyperplane that maximizes the separation of the point with the supplementary constraint of minimizing the distance ρ between the plane and the origin point.

### 3.4. Genetic algorithm

As explained in section 2.1, the classification task consists in finding at least one point *f*^+^(*t*_*l*_) from each *p*^+^ pattern that can be linearly separated from all the points *f*^−^(*t*_*l*_) of the *p*^−^ patterns. A genetic algorithm was used to test different combinations of *f*^+^(*t*_*l*_) when several *p*^+^ patterns have to be separated from background ones (*p*^−^).

For this genetic algorithm, a genotype *g* is a tuple of cardinality |*G*| = |*P*^+^| in which each element (each gene) is one *f*^+^(*t*_*l*_) belonging to each different *p*^+^ pattern. A population is composed of *M* genotypes (we use *M* = 8). For each genotype *g* of the population, a separation hyperplane is calculated to try to separate all the points *f*^−^(*t*_*l*_), *p*∈ *P*^−^ from the points *f*^+^(*t*_*l*_) ∈ g. The associated fitness function is

$$\begin{array}{l} \text{Fit}(G)=\min(\max(D_{N}(f^{+}(t_{l}))),-\max(D_{N}(f^{-}(t_{l}))))\\ \text{ with }f^{+}(t_{l})\in g\text{ and }f^{-}(t_{l})\in P^{-} \end{array}$$

Genotypes of the first generation are uniform random samples. After all genotypes of a population have been evaluated with the fitness function, the worse-performing half of the genotypes is discarded and replaced by randomly chosen new ones. The next quarter is used for reproduction, i.e., each pair of solutions is mixed by randomly interchanging one part of their genotypes to create two new solutions. Genotypes of the best quarter are used for mutation, i.e., randomly modifying the *t*_*l*_ of one gene (*f*(*t*_*l*_)) by a uniform pull in the discrete range [*t*_*l*_ − 5Δ, *t*_*l*_ + 5Δ]. In section 2.2, to avoid a bias of any one algorithm in terms of learning cycles, we took the number of hyperplanes calculated by the voltage-margin Tempotron learning rule as the maximum number of genotypes tested by the genetic algorithm. A description of the algorithm is given here in list form:

1. Initialize the first generation by a random uniform sample
2. Evaluate the fitness function for all genotypes of this generation
3. Classify genotypes according to their fitness
4. Apply mutation and reproduction to the best 50% of all genotypes
5. Replace the worse-performing 50% of the genotypes by randomly chosen new ones
6. If the maximal number of genotypes to test is not reached, go to step 2.

## 4. Discussion

In this paper, we have described a new bio-inspired method to classify spike patterns that provides better generalization results than the original Tempotron. Classification results of our method are both robust and easy to interpret in terms of neural networks. This can be interesting in electrophysiological data analysis where reliable spike burst classification is required. Moreover, because the algorithm is based on causal kernels, it can be adapted to online spike pattern classification.

Based on a geometric representation, we have obtained an analysis of the problem that reveals an intricate relation between PSP dynamics and classification capabilities of a Tempotron-like neuron. This new insights allow us to come up with a simple method to characterize computational tasks that different neuron types can solve. It might be interesting to further explore the relation between the statistics of input spike patterns (e.g., number of afferent neurons, oscillation frequencies in the afferent network) and the parameters of PSPs dynamics in the output neuron.

We introduced PSP kernel functions to support fast computations with the SVM-PSP method. In particular, the RC kernel provides good generalization performance. It might be used in electronic devices such as VLSI boards where the pulse-coded input is first transformed by a simple RC low-pass filter, while the output is compared to hyperplane equations at the start and at the end of each logical squared pulse (Mitra et al., 2009).

### 4.1. Possible improvements of the SVM-PSP method

In this paper, simulations have been performed with only one spike per neuron per pattern. However, the SVM-PSP method is not theoretically limited to such patterns. Receiving several spikes per neuron modifies the maximal values reached in each dimension of the feature space. The re-scaling for SVM computation and the distance *D*_*N*_(*t*_max_) have to be changed accordingly. In section 2.2 we have shown that optimizing the *D*_*N*_ measure is better than *M*_*V*_ for generalization purposes. Alternative notions of distance could lead to even better generalization results. These two aspects need some additional study in the future.

Furthermore, we have shown that using a genetic algorithm leads to better generalization results than the learning rule originally described along with the Tempotron, and enhanced with an increased voltage margin. However, the comparison has here been done on an easy task (i.e., separate 2 *p*^+^ pattern from 4 *p*^−^ pattern with one spike from 10 neurons). It is not clear though whether the genetic algorithm can find a good separation in more difficult tasks where more patterns have to be separated.

Tempotron-like algorithms have the advantage of using a gradient descent of a cost function whereas a genetic algorithm performs a stochastic search. A hybrid solution to solve the optimization problem would consist in running a Tempotron-like learning rule to find a separation hyperplane according to the equation *w*_1_ *V*_1_ + *w*_2_ *V*_2_ + ··· + *w*_*N*_ *V*_*N*_ − θ = 0. The times *t*^+^_max_ of maximum voltages corresponding to all *p*^+^ patterns are kept and used as seed points for the SVM-PSP method to obtain better separation coefficients (synaptic weights) for generalization.

The chosen hyper-parameters of the SVM (solver type, cost, and epsilon) provide satisfactory generalization results compared to the Tempotron and the voltage-margin Tempotron as shown in Figure 2. Fine-tuning them with a grid-based search would only improve the generalization results in our case. This kind of tuning is very-likely needed for more difficult classification tasks. We checked if those parameters modify the shape of curves of Figure 4. We used a grid-search with a cost parameter value from 10, 10000, 10000000 and an epsilon value from 0.01, 0.00001, 0.00000001 but we did not find any noticeable change in the shape (data not shown).

### 4.2. Possible improvements of the tempotron method

As previously mentioned, the training procedure of the original Tempotron algorithm stops without optimizing the separation hyperplane for generalization. To overcome this limitation, a standard procedure would consist in generating a number of jittered training samples to learn the optimal weights. But this method entails some drawbacks. First, it is difficult to find the optimal amplitude of the noise injected to generate the training sample: using a too low noise level would not lead to a better separation hyperplane. Using a too high noise level could render the problem not linearly separable, which leads to bad classification results. Secondly, the multiplication of training patterns leads to an increased computational load. The SVM-PSP method avoids these problems by directly optimizing the margin between the points and the separation hyperplane. Using jittered training samples, the difference in the performance of the standard Tempotron and the voltage-margin Tempotron would become negligible only when the maximum voltage-margin is very small, close to compromising linear separability. In this case, the SVM-PSP method would theoretically also lead to the same separation hyperplane.

Changing the kernel from the curved double exponential kernel to a kernel that generates linear piece-wise trajectories in the phase space (such as the RC kernel) does not affect the general scheme of the discretized Tempotron learning rule. By using the RC kernel, the time discretization is simplified. Times that need to be computed are *V*_*p*_ = {*V*(*t*_*ij*_), *V*(*t*_*ij*_ + *T*_pulse_)}. This can lead to a drastic reduction of computation time, especially in the case of sparse activity. The non-discretized version of the Tempotron also suggests a good method to decrease the number of relevant points by analytically resolving the times of maximum voltage between successive incoming spikes. It can be applied to double-exponential kernels, which are more similar to PSP in real nerve cells than “linear” kernels, such as the RC kernel. However, at each synaptic weight modification, and for each spike of each pattern, the non-dicretized version of the Tempotron requires the estimation of four parameters: the voltage, the synaptic current, the time of maximum voltage between two consecutive spikes, and the maximum voltage at this time. With the RC kernel for which kink times are fixed, at each synaptic weight modification, and for each spike of each pattern, only two numbers need to be determined: the voltage value at the two kink times.

One important question is whether the simple Tempotron-like neuron model is complex enough to also reflect the behavior of biological neurons. One of the most problematic differences is that biological synapses can not change their nature from excitatory to inhibitory, and *vice versa*. Another characteristic difference is that inhibitory synapses might have different kinetics than excitatory synapses (Karnup and Stelzer, 1999). Whereas with the SVM-PSP method it seems impossible to deal with these issues, one can partly resolve them by slightly modifying the Tempotron learning rules. Synaptic weights have to be initialized with the right sign, and the learning rule is modified preventing a synaptic weight to change its sign. Thus, it is possible to fix different PSP kernels for excitatory and inhibitory synapses.

### Conflict of interest statement and Publication Transfert

The authors declare that the research was conducted in the absence of any commercial or financial relationships that could be construed as a potential conflict of interest. None of the material in this paper has been previously published or is under consideration for publication elsewhere.

## Appendix

### A.1. Normalized orthogonal distance between a point and the synchrony vector in the feature space

Let us consider the point *f*(*t*) = (*f*_1_(*t*), …, *f*_*N*_(*t*)) in the feature space *S*. *f*_*s*_ = (1, …, 1) denotes the synchrony vector, *f*'(*t*) is the orthogonal projection of the point *f*(*t*) onto the vector *f*_*s*_, and *l*_*s*_(*t*) is the length of the vector *f*(*t*) − *f*'(*t*) normalized by $\|f_{s}\|=\sqrt{N}$. *L*_*s*_ is the maximum of *l*_*s*_(*t*) computed at all incoming spike times of one particular pattern. We found

$$\begin{array}{l} f^{\prime }(t)=\langle f(t),\;f_{s}\rangle /{\left\|f_{s}\right\|}^{2}f_{s}\\ \;=(\bar{f_{i}}(t),\;\ldots ,\;\bar{f_{i}}(t))\\ \;l_{s}(t)=\left\|f(t)-f^{\prime }(t)\right\|/\left\|f_{s}\right\|\\ \;=\sqrt{\frac{1}{N}\sum_{i}{\left(f_{i}\left(t\right)-\bar{f_{i}}\left(t\right)\right)}^{2}}\\ \;=\text{std}\left(\left\{f_{i}\left(t\right)\right\}\right)\\ \;L_{s}=\max\left(\text{std}\left(\left\{f_{i}\left(t\right)\right\}\right)\right) \end{array}$$
